# Association of HIV-infection, antiretroviral treatment and metabolic syndrome with large artery stiffness: a cross-sectional study

**DOI:** 10.1186/s12879-018-3637-0

**Published:** 2018-12-29

**Authors:** Titus F. Msoka, Gary P. Van Guilder, Yvo M. Smulders, Marceline van Furth, John A. Bartlett, Michiel A. van Agtmael

**Affiliations:** 1 Department of Internal Medicine, Kilimanjaro Christian Medical Centre, Moshi, Tanzania; 2 Department of Health and Nutritional Sciences, South Dakota State University, Brookings, SD USA; 3 VU University Medical Center, Amsterdam, The Netherlands; 4 Duke University Medical Center, Durham, North Carolina USA

**Keywords:** Metabolic syndrome, Pulse wave velocity, Augmentation index, Cardiovascular risk, First-line combined antiretroviral therapy

## Abstract

**Background:**

Effective combined antiretroviral therapy (cART) has improved life expectancy among people living with HIV-1 infection. Treated HIV-1infection increases the prevalence of metabolic syndrome (MS). Despite sub-Saharan Africa having among the highest rates of HIV-1 infection, the effects of MS in HIV-1-infected individuals on cardiovascular risk is poorly explored. The aim of the study was to assess whether MS and/or HIV-1 treatment correlates with large elastic artery stiffness in HIV-1-infected patients treated with first-line cART.

**Methods:**

The study sample comprised of 102 subjects free of cardiovascular disease and major risk factors divided into two groups based on HIV-1 infection, treatment, and MS status: HIV-1^+^/cART^+^/MS^+^ (*n* = 12); HIV-1^+^/cART^−^/MS^+^ (*n* = 16); HIV-1^−^/ MS^+^ (*n* = 10); HIV-1^+^/cART^+^/MS^−^ (*n* = 42); HIV-1^+^/cART^−^/MS^−^ (*n* = 32); HIV-1^−^/ MS^−^ (*n* = 39). MS was established according the International Diabetes Federation definition. Large artery stiffness was measured using applanation tonometry to assess aortic pulse wave velocity (aPWV) and aortic augmentation index at heart rate of 75 bpm (AIx@HR75). cART included lamivudine/zidovudine and nevirapine or efavirenz.

**Results:**

The prevalence of MS in the HIV-1-infected patients was 28%. There were no significant differences in aPWV in the non-MS groups. However, in subjects with MS, aPWV was significantly higher in the HIV-1 cART patients (9.0 ± 1.9 m/s) compared with both controls (7.5 ± 1.8 m/s; *P* = 0.018) and untreated HIV-1 patients (7.7 ± 1.3 m/s; *P* = 0.023), and these differences remained after adjustment for blood pressure and sex. Aortic PWV was significantly elevated (*P* = 0.009) in HIV-1 cART patients with MS compared to their counterparts without MS. Untreated HIV-1 patients with MS also demonstrated increased aPWV compared to their counterparts without MS (*P* = 0.05). Aortic AIx@HR75 was, on average, ~ 5% higher in HIV-1 cART patients with MS (28.3 ± 62% compared with untreated HIV-1 patients with MS (23.5 ± 9%; *P* = 0.075). Sub-group multivariate analysis identified MS as an independent predictor of increased aPWV in HIV-1 cART patients.

**Conclusions:**

Our study established that presence of MS in HIV-1 patients on treatment was associated with increased aPWV and hence increased arterial stiffness in sub-Saharan African HIV-1 patients on first-line cART.

## Background

Effective use of combined antiretroviral therapy (cART) has improved life expectancy among people living with HIV-1infection [1]. Despite immunological modulation, aging with HIV-1 infection is linked with an increased prevalence of metabolic syndrome (MS), both in the absence and presence of cART, which can accelerate the development of vascular abnormalities, particularly endothelial dysfunction [2] and increased carotid-artery media thickness [3]. In fact, large elastic artery stiffness, a marker of heightened cardiovascular risk and an independent predictor of adverse vascular events [4], is elevated in HIV-1-infected patients in North America and Europe [5] and appears to be further increased the presence of cART [6, 7]. We have previously shown that first-line cART with non-nucleoside and nucleoside reverse transcriptase inhibitors and time since HIV-1 diagnosis were independent predictors of increased large elastic artery stiffness in sub-Saharan African HIV-1-infected patients [8], largely free of clustered cardiometabolic risk factors that comprise MS. Data are limited and findings have been inconsistent in sub-Saharan African HIV-1-infected patients [9, 10], even though antiretroviral treatment and arterial stiffening could contribute to accelerated atherosclerotic disease. It is possible that the inconsistencies arise in part because HIV-1 infection and cART are both associated with a spectrum of metabolic complications such as dyslipidemia, insulin resistance, changes in body fat distribution, and increased blood pressure [9], which can impact arterial stiffening [11]. Despite sub-Saharan Africa having the highest rates of HIV-1 infection [10], MS in relation to arterial stiffness in HIV-1-infected patients is poorly explored.

The present study aimed to determine: 1) if MS is associated with increased large elastic artery stiffness in HIV-1-infected patients; and 2) if HIV-1 treatment with combination non-nucleoside and nucleoside reverse transcriptase inhibitors is associated with increased arterial stiffness compared to untreated patients.

## Methods

### Study participants

One-hundred twenty-seven HIV-1-infected adult patients (52 treatment-naïve and 75on cART) were recruited from the Infectious Disease Clinic (IDC) of the Kilimanjaro Christian Medical Center (KCMC) in northern Tanzania to participate in this cross-sectional comparative hospital-based study. KCMC is a referral hospital in the northern zone in Tanzania serving ~ 13 million patients. The IDC provides care to approximately 2000 HIV-1-infected patients free of charge with services including, but not limited to, counseling, testing and treatment. The process for recruitment and screening of the participants and the inclusion/exclusion criteria have been described elsewhere [8].

The final study sample included 102 HIV-1 patients: 48 untreated HIV-1-infected (32 without MS; 16 with MS) and 54 HIV-1-infected patients on cART (42 without MS; 12 with MS). MS was established according the International Diabetes Federation (IDF) ethnic-specific definition [11]. Antiretroviral regimens used by HIV-1 patients on cART have been described elsewhere [8]. Forty-nine HIV-1 uninfected adults (39 with MS, 10 without MS) served as controls. KCMC hospital staff and surrounding community were recruited to serve as uninfected controls and were well matched to the HIV-1 patients in terms of age and BMI. The absence of plasma HIV-1 RNA was confirmed in control subjects via real time PCR. Control subjects were screened via medical history and physical examination by medical staff affiliated with KCMC; those that presented with hepatic, renal, haematological, or cardiovascular disease, stroke, or diabetes were excluded. None of the study participants smoked. Informed consent was requested and given by all study participants. Study approval was provided by the Institutional Ethics Committee at KCMC via ethical clearance number 382. Study experiments conformed to the Helsinki Declaration.

### Body composition, metabolic and large artery stiffness measurements

The methods to measure body mass, height, abdominal obesity, percent body fat, fasting plasma lipid, lipoprotein, glucose concentrations, CD4^+^ T cell count at the time of cART initiation, estimation of percent fat, blood pressure and large artery measurements have been described previously [8].

### Statistical analysis

Between-groups ANOVA method was used to determine any differences between subject characteristics. Group differences in aPWV andAIx@HR75 were determined by General Linear Model univariate ANOVA. When indicated by a significant F value, a post-hoc test using the Tukey HSD method was performed to identify specific group differences. Velocity values were adjusted for mean arterial pressure since it is well established that arterial pressure may be a confounder of increased arterial stiffness [12] and should be taken into account when interpreting aPWV [13]. Details of statistical analyses performed have been described elsewhere [8]. MS, age, sex, and presence of cART were included as dummy (dichotomy) explanatory variable in the multiple regression model. IBM SPSS Statistics for Windows software (version 24; IBM Corp., Armonk, N.Y., USA) was utilized for statistical analyses.

## Results

Participant characteristics according to sub-groups are shown in Table 1. The point prevalence of MS in the HIV-1 patients was 28%. Subjects without MS were generally well matched, however, HIV-1-cART patients had lower body mass and BMI compared with corresponding group controls. As expected, body mass, BMI, percent body fat, and waist circumference were higher (all *P* < 0.05) in subjects with MS compared to corresponding between-group subjects without MS. There were no significant differences in indices of adiposity, blood pressure, or plasma lipids, lipoproteins, and glucose among the controls, HIV-1 untreated patients, and HIV-1-cART patients with MS.

**Table 1:** Patient characteristics with and without metabolic syndrome

	No Metabolic Syndrome			Metabolic Syndrome		
	(*N* = 113)			(*N* = 38)		
Variable	Controls (*n* = 39)	HIV-1 untreated (*n* = 32)	HIV-1 cART (*n* = 42)	Controls (*n* = 10)	HIV-1 untreated (*n* = 16)	HIV-1 cART (*n* = 12)
Age, year	45 ± 3	44 ± 6	46 ± 5	45 ± 4	47 ± 5	47 ± 4
Men, %	69	28	29	50	13	25
Body mass, kg	67.8 ± 11.8	64.7 ± 9.7	58.5 ± 11.5^*a*^	81.1 ± 10.6^*c*^	71.2 ± 12.7^*c*^	75.0 ± 15.6^*c*^
BMI, kg/m^2^	24.8 ± 4.2	24.3 ± 3.9	22.5 ± 4.1^*a*^	30.4 ± 4.6^*c*^	26.7 ± 3.2^*c*^	28.9 ± 4.1^*c*^
Body fat, %	32 ± 9	35 ± 7	33 ± 8	38 ± 5^*c*^	40 ± 4^*c*^	40 ± 4^*c*^
Waist circumference, cm	85 ± 10	81 ± 7	81 ± 11	95 ± 8^*c*^	92 ± 9^*c*^	98 ± 6^*c*^
Systolic BP, mmHg	124 ± 12	126 ± 9	120 ± 11	135 ± 7^*c*^	135 ± 13^*c*^	131 ± 18^*c*^
Diastolic BP, mmHg	74 ± 7	76 ± 5	74 ± 8	79 ± 3^*c*^	79 ± 6	79 ± 6^*c*^
LDL-cholesterol, mmol/l	2.9 ± 0.9	2.5 ± 0.8	2.7 ± 1.0	3.1 ± 0.7	2.8 ± 1.1	3.2 ± 0.9
HDL-cholesterol, mmol/l	1.2 ± 0.4	1.1 ± 0.5	1.4 ± 0.6^*b*^	1.0 ± 0.6	0.8 ± 0.3	1.1 ± 0.3
Triglycerides, mmol/l	1.1 ± 0.7	1.0 ± 0.4	1.4 ± 1.1	2.3 ± 1.5^*c*^	1.3 ± 0.6	2.0 ± 1.2
Glucose, mmol/l	4.7 ± 0.6	4.5 ± 0.7	4.8 ± 0.6	5.0 ± 0.7	4.8 ± 0.9	4.7 ± 0.7
CD4 at enrollment, cells/μL	–	665 ± 142	289 ± 46^*b*^	–	666 ± 120	302 ± 41^*b*^
CD4 (most recent), cells/μL	–	493 ± 250^*d*^	494 ± 229^*d*^	–	430 ± 255^*d*^	480 ± 189^*d*^
Months of HIV-1 infection, median	–	21.5	29.3^*b*^	–	20.6	39.8^*b*^
Months cART, median	–	–	24.0	–	–	30.9

Values are means ± *SD* unless otherwise noted. *BMI* body mass index, *BP* blood pressure, *LDL* low-density lipoprotein, *HDL* high-density lipoprotein, *cART* combined antiretroviral therapy, ^*a*^*P* < 0.05 vs. within-group controls, ^*b*^*P* < 0.05 vs. within-group HIV-1 untreated patients, ^*c*^*P* < 0.05 vs. corresponding group without metabolic syndrome, ^*d*^ *P* < 0.01 vs. within-group CD4 at enrollment

No differences were seen in the most recent CD4+ T cell counts between untreated and cART HIV-1 patients regardless of MS. There were no significant differences in the duration of HIV-1 infection between untreated and cART HIV-1 patients with MS compared to those without. There were no differences in the median duration of cART between the HIV-1 patients with MS (24 months; IQR: 15.9 to 42.3 months, *P* = 0.07) compared with HIV-1 patients without MS (30.9 months; IQR: 23.2 to 66.4 months, Table 1).

## Hemodynamics

Hemodynamic results from the time of pulse wave analysis are displayed in Table 2. Peripheral systolic and mean arterial pressures and pulse rates were higher, and peripheral diastolic pressure lower in comparison to central values in both the MS group and in patients without MS (*P* < 0.001 for all). No differences were seen among the groups in central systolic, diastolic, mean arterial, or pulse pressures. Similarly, corresponding peripheral blood pressures were not different among the groups.

**Table 2:** Central and peripheral hemodynamic data during pulse wave analysis in patients with and without metabolic syndrome

	No Metabolic Syndrome			Metabolic Syndrome		
Variable	Controls	HIV-1 untreated	HIV-1 cART	Controls	HIV-1 untreated	HIV-1 cART
	(*n* = 39)	(*n* = 32)	(*n* = 42)	(*n* = 10)	(*n* = 16)	(*n* = 12)
Central systolic BP	113 ± 12	116 ± 10	114 ± 10	126 ± 7^*c*^	120 ± 13	110 ± 11^*b*^
Peripheral systolic BP	123 ± 13^*a*^	127 ± 11^*a*^	124 ± 11^*a*^	135 ± 7^*ac*^	129 ± 11^*a*^	120 ± 13^*a*^
Central diastolic BP	75 ± 7	78 ± 6	78 ± 8	81 ± 3^*c*^	78 ± 6	74 ± 7^*b*^
Peripheral diastolic BP	74 ± 7^*a*^	77 ± 6^*a*^	76 ± 8^*a*^	79 ± 3^*ac*^	72 ± 7^*a*^	72 ± 7^*ab*^
Central MAP	87 ± 8	91 ± 7	90 ± 9	96 ± 3^*c*^	92 ± 7	86 ± 8^*b*^
Peripheral MAP	90 ± 8^*a*^	93 ± 7^*a*^	92 ± 8^*a*^	98 ± 2^*ac*^	94 ± 7^*a*^	87 ± 8^*b*c^
Central PP	38 ± 9	38 ± 8	36 ± 7	45 ± 8^*c*^	41 ± 9	36 ± 8
Peripheral PP	49 ± 10^*a*^	50 ± 10^*a*^	47 ± 7^*a*^	56 ± 9^*a*^	52 ± 9^*a*^	45 ± 8^*ab*^
PP amplification (ratio)	1.33 ± 0.17	1.33 ± 0.14	1.32 ± 0.13	1.24 ± 0.12	1.29 ± 0.12	1.25 ± 0.19
Heart rate (beats/min)	67 ± 11	76 ± 10^*b*^	74 ± 11^*b*^	66 ± 7	74 ± 6	78 ± 13^*b*^

Values (mmHg) are means ± SD. *cART* combined antiretroviral therapy, *BP* blood pressure, *MAP* mean arterial pressure, *PP* pulse pressure, ^*a*^*P* < 0.001 for within-group central variable, ^*b*^*P* < 0.01 versus within-group controls, ^*c*^*P* < 0.001 vs. no metabolic syndrome controls

### Aortic pulse wave velocity in the cohort of HIV-1 patients

There were no differences in aPWV between all HIV-1 seropositive patients (untreated grouped with cART patients) compared with seronegative control patients (data not shown). Similar to our earlier publication [8], HIV-1 cART patients (7.9 ± 1.9 m/s; *P* = 0.008) demonstrated elevated aPWV compared with untreated HIV-1 patients (7.1 ± 1.2 m/s).

### Aortic pulse wave velocity in patients with metabolic syndrome

**Fig. 1.**
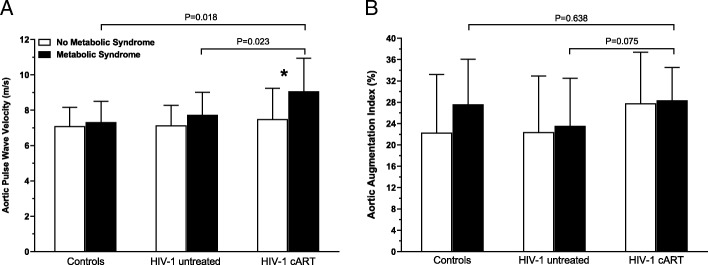
Blood pressure- and sex-adjusted aortic pulse wave velocity (**a**) and aortic augmentation index (**b**) in the controls, HIV-1-naïve and HIV-1-cART patients with and without metabolic syndrome. Aortic pulse wave velocity was 25%(*P* = 0.018) and 21% (*P* = 0.023) higher in the HIV-1 cART patients compared with both controls and untreated HIV-1 patients, respectively. There was no significant main effect of metabolic syndrome on augmentation index between the three groups. Values are mean ± SD.*P = 0.009 versus HIV-1 cART patients without metabolic syndrome.

In the entire study cohort combined by MS status, subjects with MS had significantly elevated aPWV (8.3 ± 1.6 m/s; *P* < 0.001) compared with subjects without MS (7.1 ± 1.3 m/s). Subgroup analysis in subjects without MS showed that there were no significant differences in aPWV between controls, HIV-1 untreated patients, and HIV-1 cART patients (*P* = 0.464; Fig. 1A). However, there was a significant main effect of MS among the three groups (*P* = 0.008), such that in the HIV-1 cART patients with MS, aPWV was 25% (*P* = 0.018) and 21% (*P* = 0.023) higher compared with both controls and untreated HIV-1 patients, respectively. In addition, aPWV was significantly elevated (*P* = 0.009) in HIV-1 cART patients with MS compared to their cART counterparts without MS (Figure1A). The markedly higher aPWV in the HIV-1 cART patients with MS compared with the other MS groups remained after inclusion of mean arterial pressure as a covariate (P = 0.009). These differences also remained after adjustment for sex (*P* = 0.011). There was no difference in aPWV in untreated HIV-1 patients with MS (7.7 ± 1.3 m/s) compared to their untreated counterparts without MS (7.1 ± 1.6 m/s; *P* = 0.139). Similarly, aPWV was similar in controls with and without MS (*P* = 0.590).

### Aortic augmentation in the cohort of HIV-1 patients

There were no differences in AIx@HR75 between all HIV-1 seropositive patients (untreated grouped with cART patients) compared with seronegative control patients (data not shown). Similar to our earlier publication [8], HIV-1 cART patients (27.8 ± 9%; *P* = 0.005) demonstrated elevated AIx@HR75 compared with untreated HIV-1 patients (22.7 ± 10%).

### Aortic augmentation in the patients with metabolic syndrome

There was no main effect (*P* = 0.338) of MS with respect to aortic AIx@HR75 when the study cohort was combined by MS status. Likewise, subgroup analysis in subjects without MS showed no differences in aortic AIx@HR75 between controls, HIV-1 untreated patients, and HIV-1 cART patients (*P* = 0.076; Fig. 1B) after sex adjustment. In addition, there were no differences in AIx@HR75 (*P* = 0.092) among the groups with MS. In contrast to the aPWV data, aortic AIx@HR75 was not different (*P* = 0.892) in HIV-1 cART patients with MS (28.3 ± 6.2% compared to their cART counterparts without MS (27.7 ± 9.7%; Fig. 1B).

### sIndependent predictors of large elastic artery stiffness

To identify variables that were independent predictors of aPWV in the combined sample of HIV-1 patients, we performed stepwise multiple linear regression analyses. MS, age, sex, presence of cART, included as a dummy explanatory variable (yes or no), duration of cART, body mass, LDL-cholesterol, duration of HIV-1 infection, CD4^+^ T cells at enrolment and most recent, viral load, and mean arterial pressure were included in the stepwise regression. In Table 3 a prediction model is displayed, with statistical significance (*F* = 9.901, *p* < 0.0001) and it accounted for 29% of the variance of aPWV (R^2^ = 0.290, Adjusted R^2^ = 0.261). MS (β = 0.220), age (β = 0.235), duration of cART (β = 0.275), and mean arterial pressure (β = 0.222) independently predicted aPWV, with presence of MS a stronger predictor. After including additional covariates (sex, LDL and HDL-cholesterol, triglycerides, and anthropometrics), the independent contributions of the predictors remained significant in the regression model. Examination of the structure coefficients further supports that MS was a strong determinant of aPWV among the HIV-1-infected patients. The squared structure coefficient for *MS* was 0.40, indicating that of the 29% (*R*^2^) effect, *MS independently accounts for 40% of* the explained variance in aPWV when all other predictors are held constant. Moreover, the presence of MS was independently related to a 0.8 m/s increase in aPWV after controlling for the effects of age, duration of cART, and blood pressure.

**Table 3:** Independent predictors of aortic PWV in the combined HIV-1 untreated and HIV-1 cART patients

	Predictor Variables	Multiple *R*	*R* square	Unstandardized regression coefficient (B)	95% Confidence Interval	P
Aortic PWV (m/s)	metabolic syndrome	0.341	0.116	0.816	0.135, 1.497	0.019
	Age	0.447	0.200*	0.072	0.018, 0.126	0.009
	duration of cART	0.503	0.253*	0.022	0.008, 0.036	0.002
	MAP	0.541	0.293*	0.051	0.007, 0.094	0.023

The independent predictor variables entering the final model are reported. *PWV* pulse wave velocity, *cART* combined antiretroviral therapy *Significant F change in the R square (*P* < 0.001)

To corroborate the independent effects of MS to predict changes in aPWV in the HIV-1 cART patients, we performed a separate stepwise multivariate regression analysis in this group after omitting untreated HIV-1 patients. After controlling for all potential covariates in the model, MS remained the independent predictor of aPWV (β = 0.448, *P* = 0.001) in the HIV-1 cART patients. In the HIV-1 untreated patients alone, age (β = 0.468, P = 0.001) was the only independent predictor of aPWV.

Examining AIx@HR75, MS was not a significant predictor in the combined HIV-1 group (HIV-1 untreated and HIV-1 cART). Rather, duration of cART (β = 0.288), diastolic pressure (β = 0.301), and sex (β = 0.192) independently predicted aortic AIx. The model was statistically significant (*F* = 8.041, *P* < 0.0001) and accounted for ~ 17% of the variance of aortic AIx@HR75 (*R*^2^ = 0.198, Adjusted R^2^ = 0.173).

In the uninfected control group, a stepwise multivariate regression was also performed to identify independent predictors of aPWV. After controlling for all potential covariates in the model, the only significant predictor of aPWV in the control group was diastolic pressure (β = 0.405, *P* = 0.004).

## Discussion

The main findings of the present study were that MS and other risk variables (i.e., blood pressure, duration of cART, and age) are associated with increased large elastic artery stiffness in in sub-Saharan African HIV-1-infected patients. Importantly, the degree of arterial stiffness in HIV-1 patients with MS is worse in the presence of combination non-nucleoside and nucleoside reverse transcriptase inhibitors. This suggests that perhaps treated HIV-1 infection coupled with clustering of risk factors of MS may increase aPWV and hence arterial stiffness. Increased arterial stiffness may represent an important link contributing to atherosclerotic vascular disease associated with HIV-1 infection and cART. It would benefit practitioners, and be clinically advantageous to treated HIV-1 infected individuals with MS to discriminate between those with and without increased arterial stiffness, in sub-Saharan Africa HIV-1 patients.

Our findings showed that aPWV was significantly elevated in individuals with MS compared to their counterparts without MS. Other studies also reported elevated aPWV in individuals with MS compared to those without [14, 15]. Further analysis demonstrated that HIV-1 cART patients compared to their cART-naïve counterparts had significantly higher aPWV. These findings corroborate similar studies [7, 8, 16]. HIV-1 cART with MS compared to HIV-1 cART-naïve and controls with MS had a significant elevated aPWV even after inclusion of mean arterial pressure as a covariate and after adjustment for sex. Furthermore, a significant difference was observed in arterial stiffness when HIV-1 cART patients with MS were compared to HIV-1 cART patients without MS. However, no significant elevation in aPWV was noted between untreated HIV-1 patients with MS and their counterparts without MS. Similar findings have been reported in a study done in Italy [17], though they did not find significant differences between HIV-1 cART and HIV-1 cART-naïve patients with MS. The fact that there was no difference in aPWV in untreated HIV-1 patients with MS compared to their untreated counterparts without MS would imply that cART together with MS impacts aPWV. It is argued that systemic inflammation of any cause could initiate the process of endothelial damage which is characterized by abnormal orientation of endothelial cells in the direction of blood flow in contact with the vessel wall [18]. According to Monsuez et al. [19] the molecular mechanisms by which HIV-1 induces endothelial dysfunction are multifactorial with several theories linking them with combinations of high viral load, elevated inflammatory markers and adhesion molecules, pro-atherogenic lipid profile and the effects of cART. Contrary to our findings, a study in sub-Saharan Africa found higher prevalence of MS and increased arterial stiffness in cART-naïve HIV-infected individuals compared to healthy individuals in Cameroon [20]. However, the study did not involve either HIV-infected individual on cART or stratify the groups according to MS status. Also, a review of arterial stiffness and HIV infection in adult Africans concluded that arterial stiffness is elevated in HIV-infected individuals regardless of antiretroviral treatment status [21].

Upon stepwise multivariate analysis, independent predictors of aPWV were found to be presence of MS, age, duration of cART and mean arterial pressure. Further subgroup analysis revealed that in the HIV-1 cART group, MS remained the only predictor of aPWV while in the HIV-1 cART-naïve group only age predicted aPWV. This could imply that treated HIV-1 infection coupled with the presence of MS is associated with increased aPWV and hence arterial stiffness. Similar to our study findings, the study by Maloberti et al. [17] demonstrated that pulse wave velocity was elevated in HIV patients on treatment with MS than in their counterparts without MS indicative of independent effect of MS on pulse wave velocity. Also, consistent to our study findings, Rider et al. [16] found that MS had an additive effect to HIV-1 on elevated aPWV. However, they concluded that, independent of MS, patients with treated HIV-1 have reduced vascular function and that the magnitude of the effect of treated HIV infection is the same as that of the MS. One possible explanation of the effect of cART is on lipid metabolism, endothelial and adipocyte cell function and mitochondrial dysfunction [22]. Also, cART may activate pro-inflammatory cytokines which are a result of infection and inflammation. These abnormalities are believed to lead to antiretroviral-associated metabolic syndrome [23]. Different organs and organ systems are affected by systemic complications as a result of MS. One characteristic of MS is that it is associated with alterations of the arterial vasculature, especially of the endothelium, and basal membrane and also polymorphonuclear activation, increased oxidative stress, and changes in the expression of matrixmetalloproteinases (MMPs)– family of enzymes essential for degradation of extracellular matrix during the embryonic development, morphogenesis and tissue remodeling [24, 25]. Medley et al. [26] point out that, MMP-9 in particular, could be associated with large artery stiffening and hence vascular disease. It is suggested that, the extracellular matrix of the vessel walls that are made up of collagen and elastin [27] are closely related to structural strength and elasticity of the blood vessels and are regulated by catabolic MMPs. MMPs degrade the extracellular matrix by affecting the production of weaker collagen and worn elastin fibers leading to arterial stiffening [28]. On the other hand, vascular stiffening may be a result of a complex interplay between various independent and inter-dependent factors which include age, the hormonal situation, the individual’s glycemic state and salt intake, as well as the global decline in cellular systems and function [27].

Contrary to aPWV, we did not observe differences in AIx@HR75 between HIV-1 cART patients with MS compared to their cART counterparts without MS. Several studies have shown a dissociation of aortic pulse wave velocity and augmentation index in patients with MS and healthy individuals. For example, Vlachopoulos et al. [29] found that circulating MMPs in healthy persons were inversely associated with large artery stiffness determined by aPWV, but not with wave reflections, which are measured by AIx. In their earlier randomized, double blind, sham procedure-controlled study in healthy subjects, Vlachopoulos et al. [30] found that acute systemic inflammation leads to increases large artery stiffness and a decrease in aortic AIx. Similar findings on the dissociation between aPWV and AIx were revealed in a study done by Vágovicová et al. [31] regarding the association of MS with arterial properties. They reported that aPWV was higher in subjects with MS compared with those without. In addition, aPWV was elevated in subjects with a greater number of MS risk factors. In contrast, AIx was lower in subjects with a higher number of MS risk factors, even after adjustment for age gender heart rate and mean arterial pressure. The possible explanation for the dissociation between aPWV and AIx as indicators of arterial stiffness could be that inflammation increases PWV resulting in an early return of wave reflections to central arteries. At the same time, it induces vasodilation and thus decreases the magnitude of reflected waves culminating in a neutral or decreased net result of wave reflection indices. On the other hand, Cheng and Holewijn et al. [32, 33] pointed out the dissociation could be due to the discrepancy in the AIx formula; arguing that, the AIx formula has other factors apart from central second peak and augmentation pressure and hence cannot be solely attributed to changes in central second peak and augmentation pressure. However, this needs further validation.

In this study we found the point prevalence of MS among HIV-1-infected patients was 28%. This is consistent with prevalence data reported in other studies in Tanzania [34, 35] and similar to studies in the developed world [36–38]. However, great variations in prevalence of MS have been reported in other sub-Sahara African countries ranging from 11% in Botswana [39] to 58% in Uganda [40] among self-reported adherent HIV-infected patients using at least two MS components. The differences could be attributed by the sample size and/or difference in MS criteria used. The reported high prevalence of MS in HIV-1 infected in the Ugandan study could be due to involvement of only cART-adherent study population with no substitutions which other studies do not specify.

## Clinical perspective

This study shows that metabolic syndrome (MS) in treated HIV patients further contributes to CVD risk as measured by arterial stiffness. It should make the clinician extra aware of the importance of CVRM (smoking, blood pressure, lipids, and lifestyle) in HIV patients with MS.

During screening our study excluded patients with clinical evidence of cardiometabolic disease, known to stiffen arteries. By excluding these patients we aimed at removing variables that could have directly influenced the outcome variable. The observational nature of the study limits the establishment of the direct causative effect MS on arterial stiffness. Moreover, the small sample size and even smaller sub-groups with/without MS limits the power to detect important associations as well.

## Conclusion

In conclusion, in stark contrast to HIV-1 treatment-naive patients with MS, the presence of MS was associated with more severe large elastic arterial stiffening in HIV-1 patients treated with combination non-nucleoside and nucleoside reverse transcriptase inhibitors. In addition, the large artery stiffness was greater in HIV-1 cART patients with MS compared to treated patients free of MS. These findings suggest that while cART may contribute to the development of MS, it may also exaggerate the development of arterial remodeling.

## Abbreviations

°C: degrees Celsius
Aix: Augmentation index
AIx@HR75: Aortic augmentation index at heart rate of 75 beats per minute
ANOVA: Analysis of variance
aPWV: Aortic pulse wave velocity
BMI: Body mass index
cART: Combination antiretroviral therapy
CD4^+^ T: Cluster of differentiation 4 of T cells
CVRM: Cardiovascular Risicomanagement
ECG: Electrocardiogram
HDL: High density lipoprotein
HIV-1: Human Immuno-deficiency virus type 1
HIV-1: RNA human Immuno-deficiency virus type 1Ribo-nucleic acid
HIV-1^−^/ MS^−^: HIV-1-uninfected without metabolic syndrome
HIV-1^−^/ MS^+^: HIV-1-uninfected with metabolic syndrome
HIV-1^+^/cART^−^/MS^−^: HIV-1-infected without metabolic syndrome and not on antiretroviral therapy
HIV-1^+^/cART^−^/MS^+^: HIV-1-infected with metabolic syndrome and not on antiretroviral therapy
HIV-1^+^/cART^+^/MS^−^: HIV-1-infected without metabolic syndrome and on antiretroviral therapy
HIV-1^+^/cART^+^/MS^+^: HIV-1-infected with metabolic syndrome and on antiretroviral therapy
HSD: Honest significance of differentiation
IDC: Infectious diseases clinic
IDF: International diabetes federation
IQR: Inter-quartile range
KCMC: Kilimanjaro Christian medical center
KCMC-IDC: Kilimanjaro Christian medical center- infectious diseases clinic
KCMUCo: Kilimanjaro Christian Medical University college
LDL: Low density lipoprotein
MMPs: Matrix metalloproteinases
MS: Metabolic syndrome
NIH: National Institutes of Health
NUFFIC: Netherlands Organization for International Cooperation in Higher Education
PCR: Polymerase chain reaction
US: United States
USA: United States of America
