# Correction to: Association of HIV-infection, antiretroviral treatment and metabolic syndrome with large artery stiffness: a cross-sectional study

**DOI:** 10.1186/s12879-019-3728-6

**Published:** 2019-02-22

**Authors:** Titus F. Msoka, Gary P. Van Guilder, Yvo M. Smulders, Marceline van Furth, John A. Bartlett, Michiel A. van Agtmael

**Affiliations:** 1 Department of Internal Medicine, Kilimanjaro Christian Medical Centre, Moshi, Tanzania; 2 Department of Health and Nutritional Sciences, South Dakota State University, Brookings, SD USA; 3 VU University Medical Center, Amsterdam, The Netherlands; 4 Duke University Medical Center, Durham, North Carolina USA

**Correction to**: **BMC Infectious Diseases (2018) 18:708**

**https: 10.1186/s12879-018-3637-0**

Following publication of the original article [1], the authors reported that they have provided the wrong caption. In Fig. 1 the caption should be “Blood pressure-and sex-adjusted aortic pulse wave velocity (a) and aortic augmentation index (b) in the controls, HIV-1-naive and HIV-1-cART patients with and without metabolic syndrome”.

2) The remaining sentence “Aortic pulse wave velocity was 25%( *P* = 0.018) and 21% ( *P* = 0.023) higher in the HIV-1 cART patients compared with both controls and untreated HIV-1 patients, respectively. There was no significant main effect of metabolic syndrome on augmentation index between the three groups. Values are mean ± SD. * *P* = 0.009 versus HIV-1 cART patients without metabolic syndrome” should be added to at the end of paragraph “Aortic pulse wave velocity in patients with metabolic Syndrome”.

(3) The heading “sIndependent predictors of large elastic artery stiffness” should be replaced with “Independent predictors of large elastic artery stiffness”.

It has been corrected in the original article as well.

The publisher apologizes for any inconvenience caused by this error.
